# Lycorine Pre-Treatment Alleviates Microglia Inflammation After Cerebral Ischemia by Inhibiting NF-κB Phosphorylation

**DOI:** 10.3390/brainsci15030290

**Published:** 2025-03-09

**Authors:** Wuyan Zheng, Wanyu Wu, Yuhan Li, Bo Qin, Yuping Wang, Yunhan Zeng, Betty Yuen Kwan Law, Vincent Kam Wai Wong

**Affiliations:** 1 Dr. Neher’s Biophysics Laboratory for Innovative Drug Discovery, State Key Laboratory of Quality Research in Chinese Medicine, Macau University of Science and Technology, Macau SAR, China; zhengwuyan1992@163.com (W.Z.); www.825994612@foxmail.com (W.W.); 1210013027@student.must.edu.mo (Y.L.); qinbo654@163.com (B.Q.); wyp-dy@163.com (Y.W.); kiara040816@outlook.com (Y.Z.); lykbetty@gmail.com (B.Y.K.L.); 2 The Affiliated Traditional Chinese Medicine Hospital, Southwest Medical University, Luzhou 646000, China

**Keywords:** ischemic stroke, lycorine, MCAO, inflammation, NF-ĸB pathway

## Abstract

Background: Middle-aged and elderly individuals may experience detrimental health effects due to ischemic stroke (IS). The inflammatory response triggered during IS exacerbates neuronal damage, becoming a barrier to effective IS treatment and leading to poor patient prognosis. Nevertheless, the specific role of microglia in the inflammatory response triggered by IS remains mostly unclear. The primary target of this investigation is to study the neuroinflammatory impact of lycorine (LYC) during the IS process. Our objective is to evaluate whether LYC deploys its anti-inflammatory effect with modulation of the NF-κB signaling pathway, thereby reducing IS symptoms. Methods: In this research, BV-2 cells were pre-treated with LYC for 24 h before LPS was added to induce inflammation. Results: It has been discovered that LYC suppresses BV-2 cell polarization and reduces the levels of inflammatory cytokines (IL-1β, IL-6, TNF-α), showing its potential anti-inflammatory effects in vitro. Furthermore, IκBα and p65 play crucial roles in regulating the inflammatory response within the NF-κB signaling pathway. Mechanistic exploration indicates that LYC can activate the expression of IκBα in LPS-induced BV-2 cells. IκBα inhibits NF-κB by binding to its p65 subunit, sequestering it in the cytoplasm and preventing its translocation to the nucleus, thereby inhibiting inflammation. Additionally, p65 is a key transcription factor for pro-inflammatory genes, and its downregulation leads to decreased transcriptional activity of these genes. The combined effect of increased IκBα and decreased p65 results in significantly reduced NF-κB activity, thereby alleviating the inflammatory response. Meanwhile, in vivo studies indicate that LYC pre-treatment significantly reduces the infarct size caused by middle cerebral artery occlusion (MCAO) in rats. The assessment of cerebral infarction volume, neurological scores, brain edema rate and inflammation levels in MCAO rats pre-treated with LYC indicates positive therapeutic effects. Conclusions: In summary, our research indicates that LYC pre-treatment has significant anti-inflammatory effects by attenuating inflammation levels through NF-κB inhibition, which contributes to potential therapeutic benefits in ischemic stroke (IS) and may improve disease prognosis. LYC may serve as an adjunctive clinical pre-treatment for IS, which has to be confirmed by clinical trials in the future.

## 1. Introduction

Ischemic stroke (IS), commonly referred to as cerebral infarction, is a globally prevalent condition, constituting the most typical form of stroke and liable for roughly 87% of cases worldwide [1]. IS is defined as the interruption of cerebral arterial blood flow due to various factors, leading to neurological impairments resulting from localized hypoxia and ischemic necrosis in brain tissue [2]. IS has a dismal prognosis, and most patients recover with disabilities, incurring large financial costs [3]. Therefore, developing innovative treatments to aid long-term recovery from stroke is both desirable and important. In response to ischemic seizures, damaged tissue is removed, and brain repair is aided by activated resident immune cells in the central nervous system, such as microglia and astrocytes. However, the severe inflammatory response can exacerbate neuronal injury [4]. As a result, numerous studies have shown that reducing neuroinflammation in the brain by targeting microglia and astrocytes can effectively improve the injury and prognosis of IS [5].

NF-κB signaling pathways are generally divided into two pathways, i.e., the canonical and non-canonical pathways. In the former, receptors like TNF receptor, IL-1β receptor, and antigen receptor sense stimuli such as cytokines, initiating a signaling cascade [6]. Growing evidence underscores the prominence of the NF-κB signaling pathway while expounding the pathogenesis of IS [7]. In response to changes in the microenvironment of the central nervous system, microglia can polarize to either an anti-inflammatory or a pro-inflammatory phenotype [8]. Inflammatory microglia emit cytokines and chemokines that promote inflammation and contribute to neuronal death [9]. NF-κB is a critical regulatory pathway in microglial activation and polarization, and its inhibition reduces the shift of microglia toward a pro-inflammatory phenotype [10].

Amaryllidaceae is a family of herbs widely used in traditional Chinese medicine. LYC, first isolated from Narcissus in 1877, contains lycorine as its primary active alkaloid. The chemical structure and bioactivities of lycorine have been extensively studied [11]. LYC has various pharmacological effects, with antibacterial, antiparasitic, anti-inflammatory, and antitumor properties [12]. Norbelladine, the precursor to LYC, operates as an anti-inflammatory agent by inhibiting NF-κB activation [13]. It has been showcased by research that the inflammatory response triggered by the PI3K-AKT/NF-κB pathway is inhibited by LYC, which also attenuates hypertensive heart failure in mice [14]. Stilbene has also been proven to have neurological applications, and the LYC-type alkaloid assoanine is an acetylcholinesterase inhibitor that efficiently inhibits brain cholinergic dysfunction in Alzheimer’s disease [15,16]. However, whether LYC has similar anti-neuroinflammatory effects in IS is not widely known.

Our study aims to determine if LYC’s anti-inflammatory effects are achieved via regulating NF-κB, leading to reduced IS symptoms.

## 2. Materials and Methods

### 2.1. Cell Culture and Pre-Treatment

The BV-2 microglial cells were sourced from the American Type Culture Collection (ATCC, Rockville, Bethesda, MD, USA) and grown in Dulbecco’s Modified Eagle’s Medium (DMEM, Santa Clara, CA, USA), enriched with 10% fetal bovine serum (FBS) and 1% penicillin–streptomycin. Five experimental groups were formed for the cells: a control group, an LPS group (LPS at 2 µg/mL), and three treatment groups, in which cells were pre-treated with LYC at concentrations of 1.3 µM, 2.7 µM, and 5.4 µM for 24 h, followed by LPS stimulation at 2 µg/mL for an additional 12 h. The cells were incubated at 37 °C with 5% CO_2_.

### 2.2. Cell Viability

The 3-(4,5-dimethylthiazol-2-yl)-2,5-diphenyltetrazolium bromide (MTT) assay was employed to assess cytotoxicity, with a concentration of 5.0 mg/mL. The BV-2 cells were plated in 96-well plates and left to incubate all through the night. Afterward, BV-2 cells were exposed to a series of LYC concentrations (0.10, 0.39, 0.78, 1.56, 3.13, 6.25, 12.20 μM). Following a 4-h incubation, MTT was introduced to each well in a volume of 10 μL, after which 100 μL of lysis buffer, consisting of 10% SDS in 0.01 mol/L hydrochloric acid, was drizzled. The plates were then left to incubate overnight. The optical density (OD) values were assessed at A570 nm utilizing an enzyme marker. The cell viability was assessed by utilizing the formula:

Cell viability (%) = (Treated cell number/DMSO control cell number) × 100.

### 2.3. Bright-Field Imaging

Bright-field imaging was conducted utilizing an Olympus inverted fluorescence microscope (Olympus, Tokyo, Japan) equipped with a 40x objective lens. Images were captured utilizing an IncuCyte ZOOM live cell imaging system (Sartorius, Gottingen, Germany). To ensure robust data collection, at least five randomly selected fields were imaged for each experimental condition.

### 2.4. Immunofluorescence Staining

The fixation of BV-2 cells was performed in 4% formaldehyde for 10 min, and BV-2 cells were subsequently rinsed with phosphate-buffered saline (PBS, Gibco, Vacaville, CA, USA) containing 0.1% Triton X-100 (Beyotime, Shanghai, China). Subsequently, the cells were fixed with 3% bovine serum albumin and stained with phalloidin-fluorescein isothiocyanate (Actin-tracker red, C2207s, Beyotime, China) for 15 min, and with DAPI (Solarbio, Beijing, China) for 5 min. The red fluorescence indicating cell microfilaments was visualized using a fluorescence microscope (Nikon ECLIPSE 80i, Tokyo, Japan). For each experimental condition, at least five randomly selected fields were captured to ensure comprehensive analysis.

### 2.5. Real-Time Quantitative Polymerase Chain Reaction (RT-qPCR)

The BV-2 cells were plated in 6-well plates and treated with LYC with concentrations at 1.3, 2.7, and 5.4 μM for 24 h, followed by a further 12-h exposure to LPS (2 μg/mL). For animal samples, total RNA was extracted from the left cerebral cortex tissue using TRIzol^®^ Reagent (Invitrogen, Carlsbad, CA, USA). The sampling procedure was standardized to collect tissue consistently from the same region of the left cortex, ensuring uniform sample size across all animals. Total RNA from cell samples was segregated utilizing the FavorPrep™ Total RNA Purification Mini Kit (Favorgen Biotech, Pingdong, Taiwan). The concentration of RNA was measured utilizing a NanoDrop 2000c Spectrophotometer (Thermo Scientific, Vacaville, CA, USA) prior to cDNA synthesis, which was carried out utilizing a reagent kit from Beyotime. RNA levels were quantified utilizing Real-Time PCR with a ViiA™ 7 Real-Time PCR System (Applied Biosystems, Sparta, NJ, USA) and SYBR Green (Roche Diagnostics, Indianapolis, IN, USA) according to the manufacturer’s instructions. Every sample was subjected to 45 PCR cycles, which included 35 s at 95 °C, 15 s at 55 °C, and 10 s at 72 °C. The analysis of the melting curve was performed to verify the specificity of the product. Utilizing the primers outlined in Table 1, the comparative CT techniques (2^−ΔΔCT^) were exploited to determine the relative expression levels.

### 2.6. Western Blotting

The cell protein samples were prepared according to the experimental groups and heated at 95 °C for 5–10 min prior to electrophoresis. Total proteins were extracted from brain samples using RIPA buffer to ensure effective lysis and were then quantified with the BCA assay for precise protein concentration determination. Equal amounts of proteins were combined with protein loading buffer, then separated by SDS–PAGE and transferred onto polyvinylidene fluoride (PVDF, Bio-Rad, Portland, ME, USA) membranes. The membranes were washed with TBST and blocked with 5% skim milk or bovine serum albumin (BSA) for 3 *h* to prevent nonspecific binding. Primary antibodies, including anti-NF-κB p65 (1:1000; naturebios AF5243, Hangzhou, China), anti-IκBα (1:500; Wanleibio WL01936, Shengyang, China), and anti-P-p65 (1:1000; Beyotime AF5875, Shanghai, China), were added, followed by incubation overnight at 4 °C. β-actin (diluted 1:10,000) was used as a normalization control to ensure consistency across samples. After five washes in TBST, the membranes were incubated for 1 h with either anti-mouse or anti-rabbit secondary antibodies (1:3000; Cell Signaling Technology, Danvers, MA, USA) conjugated to peroxidase. For visualization, band detection was achieved using the SuperSignal West Femto Maximum Sensitivity Substrate Kit (Thermo, Vacaville, CA, USA) and captured on the Amersham Imager 800 (GE) Imaging System, with color development achieved via electrochemiluminescence (ECL). Quantitative analysis of band intensities was performed with ImageJ software (version 1.5.4, Java software v10.0, National Institutes of Health, MD, USA) by calculating the ratio of target protein band intensity to that of β-actin.

### 2.7. Animals

All animal experiments utilized Sprague Dawley rats (n ≥ 5 rats per group), each weighing between 240 and 280 g. The animals were obtained from Macau University of Science and Technology, Macau, China. The rats were kept in separate housing on the basis of a 12-h light/dark cycle, with the temperature set at 23–27 °C and relative humidity maintained at 30–60%. They had access to food and sources of hydration and were given 2–3 days to adjust to these environmental conditions. All animal care and experimental procedures were conducted in accordance with the guidelines set by Macau University of Science and Technology.

### 2.8. Middle Cerebral Artery Occlusion (MCAO) Surgery and Treatment Protocol

After 2–3 days of acclimatization, the rats were randomly divided into four groups: sham group (n ≥ 5 rats per group, 0.9% NaCl), MCAO group (n ≥ 5 rats per group, MCAO + 0.9% NaCl), LYC 1 mg/kg group (n ≥ 5 rats per group, MCAO + LYC 1 mg/kg), and LYC 5 mg/kg group (n ≥ 5 rats per group, MCAO + LYC 5 mg/kg). LYC (purity > 98%, Shanghai Yuanye Biotechnology, Shanghai, China) was suspended in dimethyl sulfoxide (DMSO) and then diluted with a 0.9% sodium chloride solution for clinical use. Each group was intraperitoneally injected once a day for 5 days in advance, and 2–3 h before MCAO surgery at different doses of LYC or 0.9% NaCl, respectively.

For MCAO surgery, the rats were sedated using sodium pentobarbital (40 mg·kg^−1^). The intraluminal filament technique was used to induce middle cerebral artery occlusion (MCAO) for 24 h. Briefly, a midline neck incision revealed the left common carotid artery (CCA), internal carotid artery (ICA), and external carotid artery (ECA). The ICA was temporarily clipped, and the ECA and CCA were secured with silk sutures. A monofilament nylon suture was inserted into the ICA and gradually advanced to the middle cerebral artery (MCA), where it remained until sacrifice. The entire surgical procedure was completed within fifteen minutes.

### 2.9. Measurement of Cerebral Edema and TTC Staining

24 h after the MCAO, the left and right cerebral hemispheres were severed, and their moist weights were quickly ascertained by weighing them. The following formula was used to calculate the cerebral edema rate: Cerebral edema rate (%) = (Left brain weight − Right brain weight)/Total brain weight × 100% [17].

The left hemisphere was designated as the infarcted area. Brain tissues were then sectioned into six 2 mm slices for assessment of infarction areas. The parts were coated in 4% paraformaldehyde after the tissues were nursed for 30 min at 37 °C using a 2% 2,3,5-Triphenyltetrazolium chloride (TTC) (Solarbio, China) incubation.

The injury area of each section was quantified as a percentage of the ipsilateral hemisphere normalized to the contralateral hemisphere (edema correction) using ImageJ software.

### 2.10. Evaluation of Neurological Score

**Figure 1:**
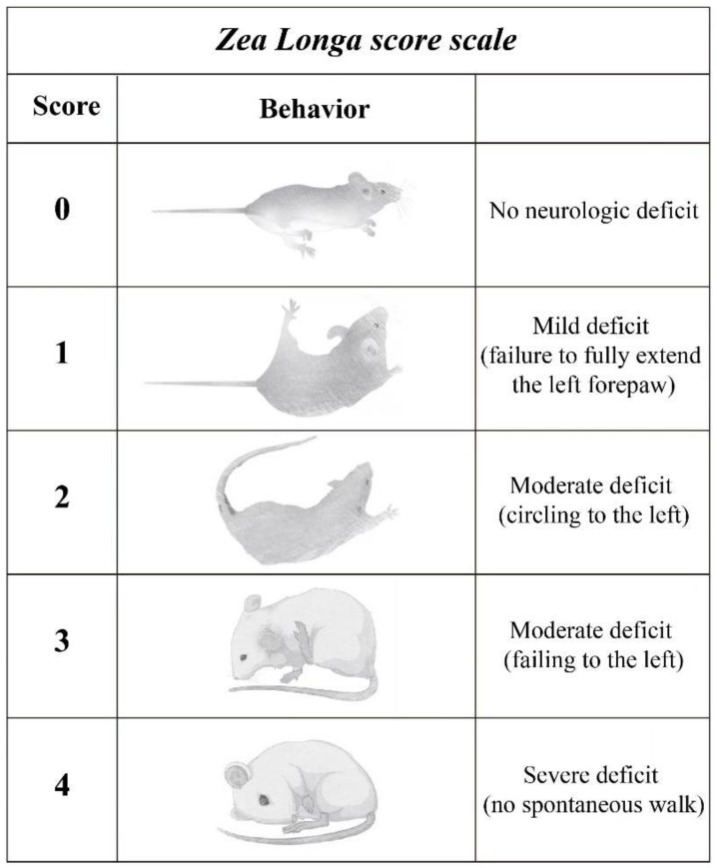
The Zea Longa scoring system was defined as follows: a score of 0 indicated no neurological deficit, 1 represented inability to extend the right forelimb, 2 indicated circling to the opposite direction, 3 reflected descending to the contralateral side at recess, 4 indicated deficiency of spontaneous motor activity.

To clarify the therapeutic action of LYC in neurological deficits caused by MCAO surgery, the rats’ neurological function was assessed based on their behavioral performance, following standardized scoring criteria. The assessment of nerve deficits was conducted 24 h post-surgery using a blinded evaluation method. Specifically, the researchers utilized the Zea Longa score scale (Figure 1) to systematically quantify the neurological deficits associated with MCAO.

### 2.11. Detection of the Concentration of Cytokine

Blood was collected from MCAO rats, and serum was obtained by centrifuging at 3500 rpm for 20 min. The serum concentrations of inflammatory and anti-inflammatory cytokines, including IL-1α, IL-1β, IL-6, IL-33, TNF-α, GM-CSF, and IL-18, were quantified using the LEGENDplex™ Rat Inflammatory Panel or a customized panel (BioLegend, San Diego, CA, USA) following the manufacturer’s instructions. Data were collected on a BD FACSAria III cell sorter (BD Biosciences, San Jose, CA, USA) and analyzed with LEGENDplex™ Data Analysis Version 8.0 software.

### 2.12. Statistical Analysis

The outcomes are shown as mean ± S.E.M. and were gathered from. at the fewest, three different experiments. GraphPad 10.0 software, one-way ANOVA, Shapiro–Wilk Test, Mann–Whitney test, Wilcoxon test and t-tests were utilized to assess statistical significance between various groups. A *p*-value was deemed statistically significant if it was less than 0.05.

## 3. Results

### 3.1. LYC Inhibits BV-2 Cell Viability and Protects Against LPS-Induced BV-2 Polarization

**Figure 2:**
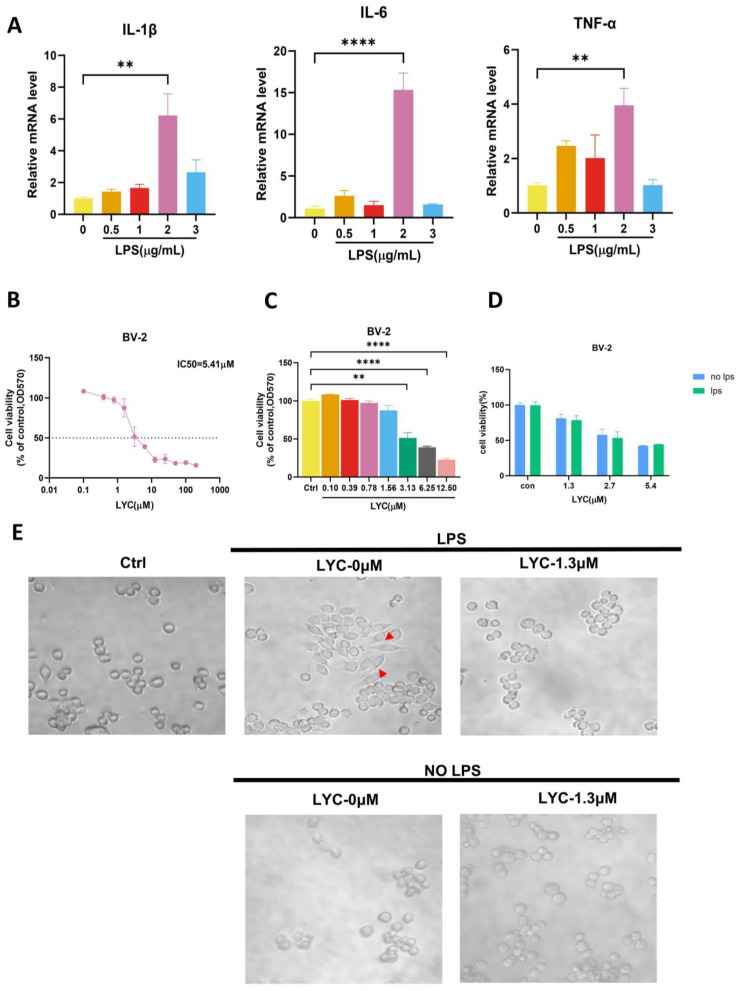

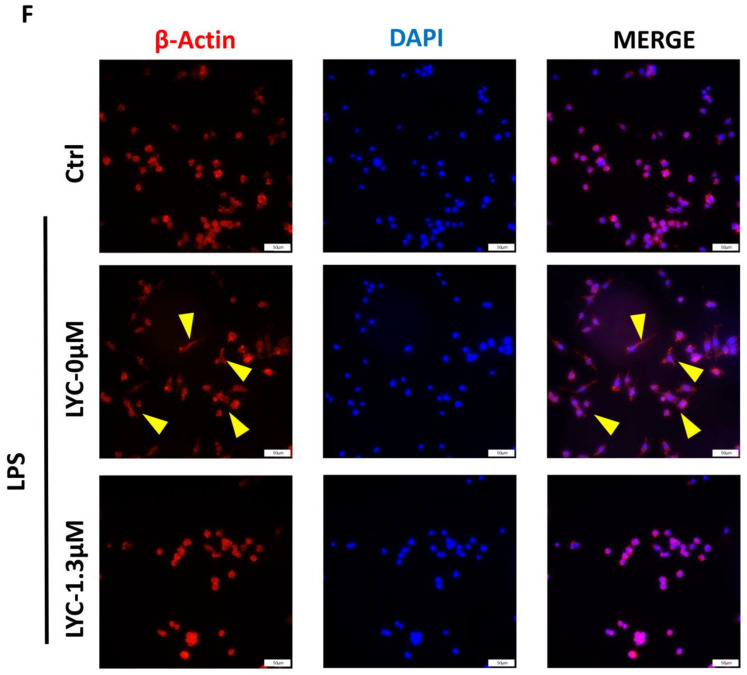
LYC inhibits BV-2 cell viability and protects against LPS-induced BV-2 polarization. LPS induces the BV-2 inflammatory model (**A**). LYC’s influence on BV-2 cell viability is assessed by using MTT test (**B**–**D**). Morphological changes induced by LYC in BV-2 cells are observed under an optical microscope (×40); red arrows indicate polarized BV-2 cells (**E**). Immunofluorescence shows LPS-induced BV-2 cell after LYC pre-treatment. TRITC (red) was used to stain the actin filaments, and DAPI (blue) was used to stain the nuclei of BV-2 cells. Yellow Arrows indicate polarized BV-2 cells. Scale bars: 50 μm. (**F**). Data are expressed as mean ± S.E.M from three technical replicates (n ≥ 3). ** *p* < 0.01 compared to control group, **** *p* < 0.0001 compared to control group.

Changes in inflammatory cytokines (IL-1β, IL-6, and TNF-α) were assessed across different treatment groups. Notably, BV-2 cells exhibit a significant increase in inflammatory factor release after stimulation with 2 μg/mL LPS (Figure 2A). Using the MTT assay, we evaluate the effect of LYC on BV-2 cell viability (Figure 2B,C). After 24 h of LYC pretreatment, BV-2 cell proliferation is inhibited, with IC50 values of 5.41 μM, indicating the concentration at which LYC inhibits 50% of cell viability, demonstrating a dose-dependent response. BV-2 cells were pre-treated with LYC for 24 h at doses of 1.3 μM, 2.7 μM, and 5.4 μM in this experiment. As a result, we confirm that BV-2 cell proliferation remains unaffected by LPS after LYC pre-treatment at different concentrations (1.3/2.7/5.4 μM) (Figure 2D). Microscopic examination reveals irregular BV-2 cell morphology with numerous pseudopodia upon LPS induction. However, after 24 h of LYC pre-treatment, activated BV-2 cells exhibit regular morphology with reduced pseudopodia (Figure 2E). The fluorescence microscopy imaging results demonstrate that BV-2 cells are stained with TRITC-labeled phalloidin (red), while the cell nuclei are stained with DAPI (blue). LPS-induced BV-2 cells exhibit significant activation, evident by pronounced cellular changes and the formation of pseudopodia (indicated by yellow arrows) (Figure 2F). Propidium iodide (PI) staining was additionally performed to confirm the absence of significant cellular mortality under LYC treatment (File S1). In contrast, LYC-treated activated BV-2 cells appear smoother and do not exhibit distinct pseudopodia. These results imply that LYC effectively suppresses BV-2 cell polarization stimulated by LPS.

### 3.2. LYC Inhibits Inflammatory Factors from Release in BV-2 Cells by Inhibiting NF-κB Pathway

**Figure 3:**
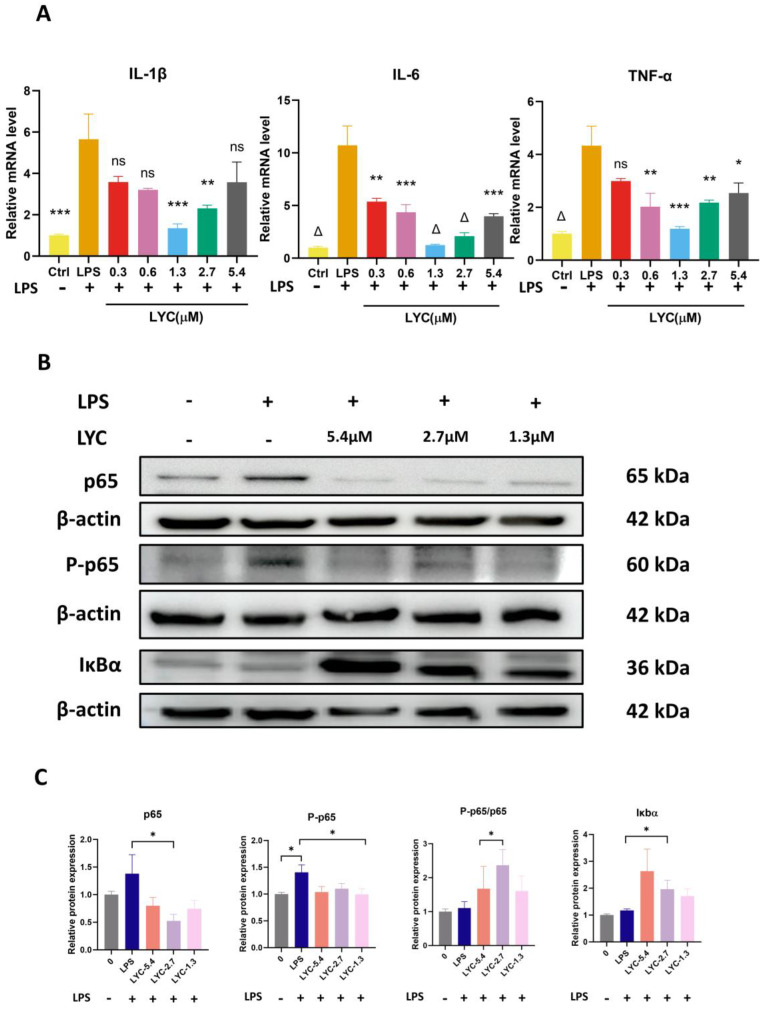
LYC suppresses the secretion of inflammatory factors in BV-2 cells by modulating the NF-κB pathway. RT-qPCR detects the anti-inflammatory effects of LPS on different concentrations of LYC-treated BV-2 cells (**A**)**.** The impact of LYC on p65/P-p65/IκBα expression in LPS-stimulated BV-2 cells, analyzed by Western blotting (B). Quantification of the blots in 3B is shown in (C). * Compared with the LPS group (*p* < 0.05), ** Compared with the LPS group (*p* < 0.01), *** Compared with the LPS group (*p* < 0.001), Δ Compared with the LPS group (*p* ≤ 0.0001), ns Compared with the LPS group (*p* > 0.05).

To verify the anti-inflammatory activity of LYC, we pre-treated BV-2 cells at varying concentrations of LYC (1.3/2.7/5.4 μM) for 24 h, followed by LPS stimulation. We evaluated the pro-inflammatory cytokine mRNA levels (IL-1β/IL-6/TNF-α) (Figure 3A). The findings indicate that, with increasing concentrations of LYC, the levels of pro-inflammatory factors are reduced to different extents. The inflammatory factors IL-1β/IL-6/TNF-α in the LPS-induced BV-2 group were significantly increased, and the level of inflammatory factors could be inhibited in the LYC 0.3μM group. When the concentration of LYC was 0.6/1.3 μm, the level of inflammatory factors was lower than that in the previous group (Figure 3A). Overall analysis indicates that 1.3 μM is the optimal anti-inflammatory concentration. (It is worth noting that 5.4 μM is the maximum concentration selected for anti-inflammatory concentration, and the anti-inflammatory results in this group may be influenced by cell death factors). After pre-treatment with LYC at different concentrations (2.7, 1.3 μM) (Figure 3B,C), the levels of IκBα protein increased, which suppress the activity of NF-Κb. Simultaneously, the levels of P-p65 decreased, resulting in the suppression of the NF-κB signaling pathway. These results indicate that the anti-inflammatory effect of LYC may be mediated through the NF-κB pathway.

### 3.3. LYC Reduces Cerebral Ischemia Injuries in MCAO Rats

**Figure 4:**
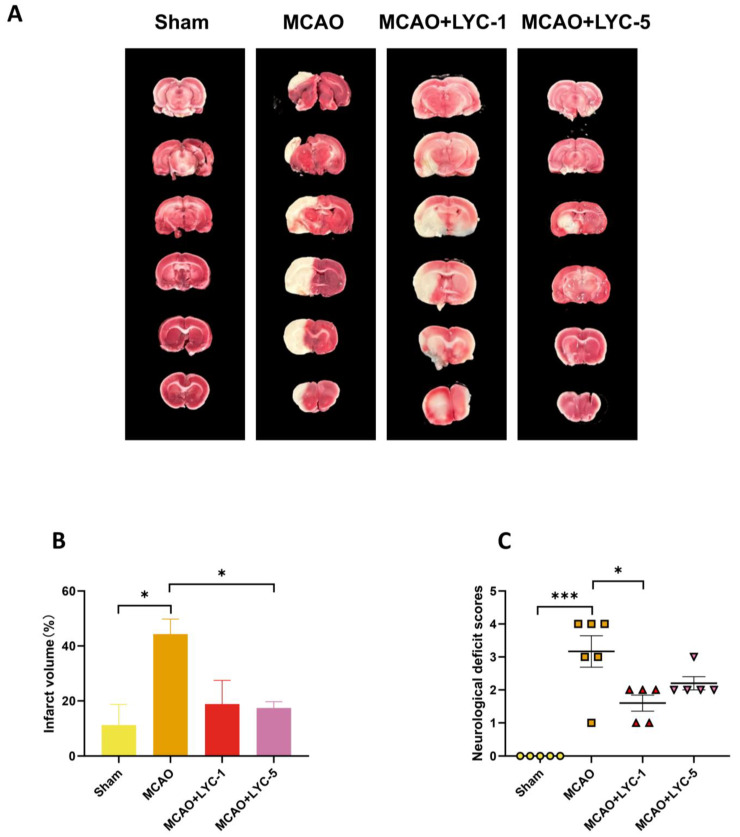
Cerebral infarct volumes are tested by 2% TTC staining (**A**,**B**). Neurological deficit scores are evaluated at 24 h after cerebral ischemia (**C**). Mean ± S.E.M. (n ≥ 5 rats per group) is used to represent the data. *** *p* < 0.001 compared to MCAO group, * *p* < 0.05 compared to MCAO group.

The present study used the MCAO model to simulate IS in vivo. After MCAO, rat survival rates are considerably lowered, and intraperitoneal administration of LYC (1 mg/kg and 5 mg/kg) has no direct effect on them (Table 2). Cerebral infarct volume is measured to assess the severity of brain damage, and LYC (1 mg/kg) reduces the MCAO-induced rise in cerebral infarct volume, but LYC (5 mg/kg) significantly inhibits the MCAO-induced increase in cerebral infarct volume (Figure 4A,B). Neurological scores are performed 24 h after surgery to assess brain injury in SD rats. The neurologic scores increased 24 h after MCAO surgery. LYC (1 mg/kg and 5 mg/kg) substantially decreases neurological scores in MCAO rats (Figure 4C).

### 3.4. LYC Suppresses the Inflammatory Response in MCAO Rats

**Figure 5:**
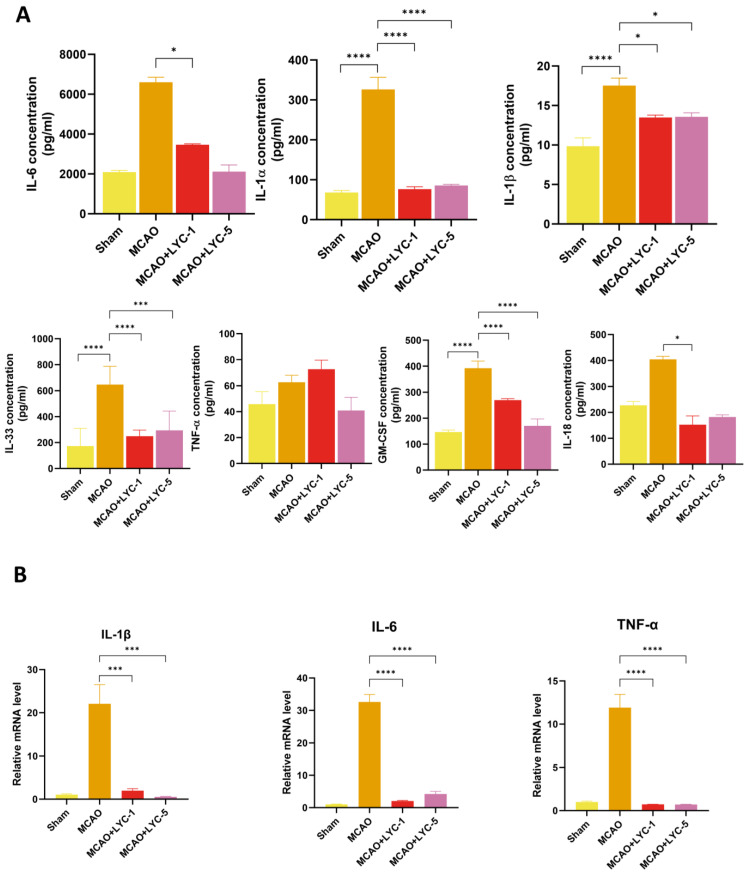
LYC attenuates inflammation response in MCAO rats. The concentration of serum caused by inflammatory cytokines in LYC-treated MCAO rats (**A**) is measured. The 3 genes related to inflammatory chemokines, IL-1β, IL-6, and TNF-α, are selected for RT-qPCR analysis (**B**). The given data is displayed as the means ± S.E.M (n ≥ 5 rats per group). **** *p* < 0.0001 compared to MCAO group; *** *p* < 0.001 compared to MCAO group, and * *p* < 0.05 compared to MCAO group.

Furthermore, we assessed the cytokine levels associated with inflammation in the serum of MCAO rats treated with LYC. In the MCAO model group, pro-inflammatory cytokine levels are noticeably higher. In particular, the concentrations of IL-6, IL-1α, IL-1β, IL-33, GM-CSF, and IL-18 are significantly elevated in the MCAO rats in comparison with the sham group (Figure 5). Administration of LYC (1 mg/kg and 5 mg/kg) significantly reverses the MCAO-induced increase in pro-inflammatory factors. Lower concentrations of IL-6, IL-1α, IL-1β, IL-33, GM-CSF, and IL-18 are detected in the serum of the LYC-pre-treatment group (1 mg/kg and 5 mg/kg) (Figure 5A).

To further elucidate the LYC-mediated anti-neuroinflammatory effects in MCAO rats, the expression of inflammatory factors was validated by real-time PCR (RT-qPCR) using brain samples. The mRNA expression levels of IL-1β, IL-6, and TNF-α were significantly elevated in the MCAO group compared to the sham group. However, these expression levels were suppressed in the LYC-pre-treated groups (1 mg/kg and 5 mg/kg) compared to the MCAO group (Figure 5B). In summary, these findings demonstrate that LYC exhibits potential anti-neuroinflammatory effects.

## 4. Discussion

LYC is an iso-quinoline alkaloid with great therapeutic potential [11,12,18]. It has high sensitivity and specific activity in vitro and in vivo against a variety of cancers and drug-resistant cancer cells and is used in a variety of inflammatory diseases [19]. It is reported that the activities of LYC are mainly in the form of antiviral [20], antibacterial [21], anti-parasitic [22], anti-inflammatory [23], and antitumor effects [24]. LYC exhibits excellent anti-inflammatory and anti-tumor effects, but it also produces central nervous system side effects, such as nausea and vomiting. Studies have indicated that the frequency of vomiting induced by LYC is dose-dependent, with symptoms of nausea and vomiting occurring within 2.5 h after administration. A dose of 30 mg/kg of LYC impairs feeding behavior in mice, increasing their immobility, which limits its clinical application. To expand the clinical application value of LYC, a comprehensive evaluation of its side effects was conducted. The study indicated that intraperitoneal injection of 10 mg/kg does not affect the motor coordination, spontaneous locomotor activity, home-cage behavior, or overall health of mice, and LYC can exert good therapeutic effects within a safe dosage range. In this study, we demonstrate that pre-treatment with LYC can alleviate symptoms of cerebral ischemia. It is observed that LYC mitigates LPS-induced polarization of BV-2 microglial cells and decreases the secretion of inflammatory factors in vitro. Furthermore, in vivo experiments demonstrate that pre-treatment with LYC can alleviate symptoms in MCAO rats and reduce inflammation levels.

In previous studies, an increasing number of experiments explored the antiviral, anticancer and anti-inflammatory effects of LYC. Among these, the combination of lycorine with NF-kB inhibitors is shown to offer a therapeutic effect against glioma by inhibiting C6 glioma cell growth and inducing cell apoptosis, as well as promoting intracellular reactive oxygen species (ROS) production [22]. LYC can inhibit the viability and migration of C6 glioma cells through the NF-κB signaling pathway. When used in combination with an NF-κB inhibitor, it can effectively exert anti-glioma effects. This confirms that the anti-inflammatory and anti-tumor effects of LYC are likely to be achieved through the NF-κB pathway. Most research focuses solely on functional recovery after cerebral ischemia, and the promising anti-inflammatory effects of LYC in treating acute cerebral hemorrhage remain unclear. Our study shows that pre-treatment with LYC inhibits cell depolarization. Therefore, we studied early ischemic infarction and found that pre-treatment with LYC can reduce ischemic infarct-related inflammation through the NF-κB pathway. In addition, pre-treatment with LYC can significantly improve the pathological and behavioral defects of brain tissue caused by cerebral ischemic infarction. Together, these findings suggest the potential clinical benefit of LYC pre-treatment in preventing or mitigating cerebral ischemic infarction.

IS, also known as cerebral infarction, is a common and prevalent disease that significantly impacts the health of elderly individuals [25]. It represents the primary type of stroke. IS occurs due to various factors that lead to interruption of blood flow in cerebral arteries, resulting in brain tissue hypoxia, necrosis, and edema. The onset of IS is rapid, with symptoms developing within minutes to hours. The inflammatory response and subsequent brain tissue damage following acute cerebral hemorrhage are critical factors affecting prognosis and outcomes [26,27].

Currently, research on the pathophysiology of IS primarily focuses on ion-level imbalances caused by hypoxic–ischemic conditions [28,29]. These imbalances lead to cell depolarization, apoptosis and subsequent neuronal damage. Additionally, early ischemic tissue in IS is linked to elevated oxidative stress, where free radicals, reactive oxygen species (ROS) and reactive nitrogen species (RNS) play key roles in cerebral ischemia [30,31]. These factors contribute to secreting pro-inflammatory cytokines, initiating a series of pathological responses and delaying disease recovery. Therefore, it is reasonable to hypothesize that mitigating inflammation in IS may improve or restore brain function and enhance IS prognosis. Inflammation is one of the earliest symptoms of acute cerebral ischemia. Quickly alleviating acute phase inflammation and preventing the formation of cerebral edema are crucial preventive measures to effectively halt the progression of cerebral ischemia. In addition to inflammation, acute cerebral ischemia in its later stages can lead to a series of symptom changes, including neurological function degradation and motor dysfunction. The treatment window for clinical acute cerebral ischemia is within a few minutes to a few hours. Therefore, rapid control of inflammation and prevention of cerebral edema are key to treating this condition. This study focuses on alleviating acute phase symptoms. Future research will further explore whether pre-treatment with LYC, in combination with other treatments, can be beneficial for the chronic treatment of cerebral ischemia and promote functional recovery. Our study shows that pre-treatment with LYC suppresses cell polarization and reduces inflammatory factor release, as evidenced by immunofluorescence and quantitative analysis. Although pre-treatment with LYC does not improve the survival rate in MCAO rats, it reduces brain infarction and alleviates inflammatory symptoms, potentially hindering IS progression. These findings suggest that pre-treatment with LYC can mitigate inflammation and improve IS symptoms.

**Figure 6:**
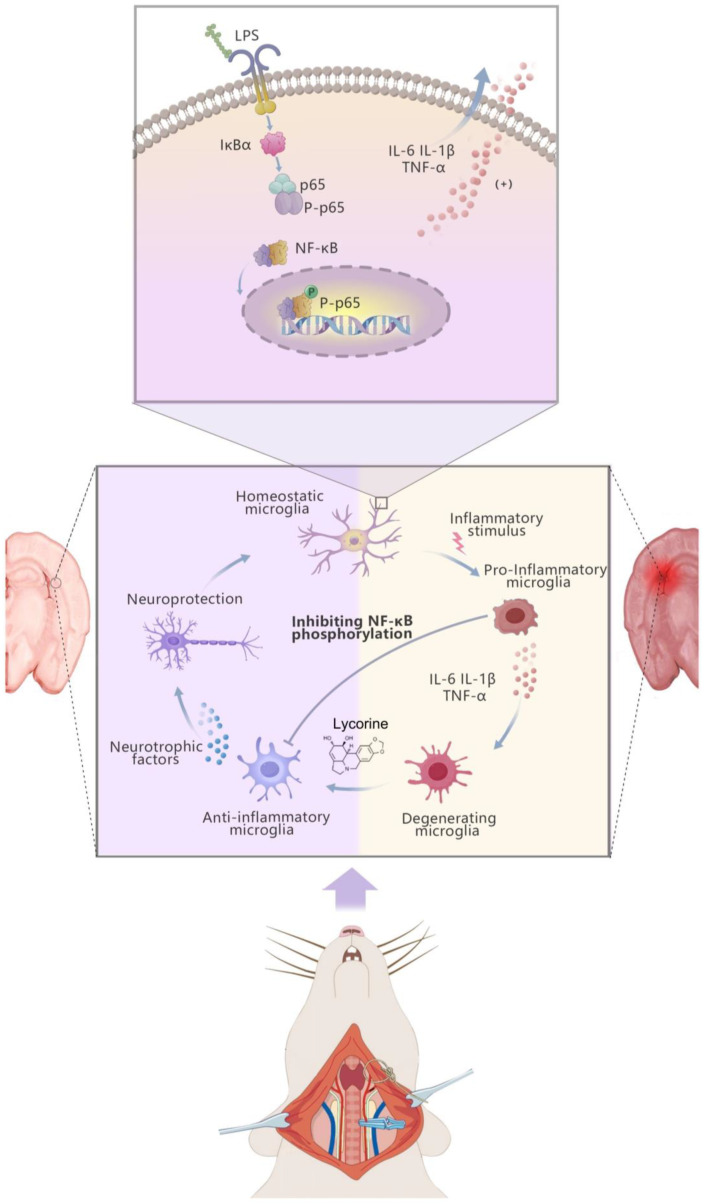
Diagram of the protective effects of LYC on ischemic brain inflammation. LYC can alleviate inflammation caused by cerebral ischemia to some extent through the NF-κB pathway.

The transcription factors that makeup NF-κB are structurally similar and have evolved to be preserved. It is involved in several processes, such as inflammation and immune regulation, cell proliferation, differentiation, and apoptosis [30]. Research reveals that NF-κB signaling performs a crucial function in the vascular system and cell types involved in thrombo-inflammatory reaction [32]. IKK family proteins and NF-κB are important components in the regulation of inflammation. The IKK family consists of IκBα/ IκBβ/ IκBγ, in which IκBα is mainly involved in the regulation of classical and non-classical NF-κB signaling pathways. In the absence of stimulation, IκBα binds to the NF-κB dimer, preventing it from entering the nucleus, thereby inhibiting its transcriptional activity. When cells are stimulated by inflammation, IκBα becomes phosphorylated and ubiquitinated, leading to its degradation and the release of NF-κB dimers, which translocate into the nucleus to activate the transcription of genes associated with inflammation. In addition, the expression of IκBα is regulated by NF-κB, forming a negative feedback loop to avoid excessive inflammatory responses. In this study, after stimulation by LPS, IκBα is resynthesized to inhibit the sustained activation of NF-κB (the expression level of the synthesized IκBα was temporarily decreased after binding with NF-κB) and prevent excessive inflammatory responses. Meanwhile, the expression of IκBα may be associated with cell apoptosis. In the future, we will further investigate the relationship between LYC-pretreated LPS-induced BV-2 cells and cell apoptosis. During inflammation, NF-κB activation leads to the upregulation of pro-inflammatory cytokines, including IL-1α, IL-1β, IL-6 and TNF-α [33]. This results in improved expression of monocyte chemoattractant protein-1 (MCP-1) and endothelial cell activation. Additionally, inflammatory factors promote the release of intercellular adhesion molecules-1 (ICAM-1), platelet endothelial cell adhesion molecule-1 (PECAM-1) and vascular cell adhesion molecule-1 (VCAM-1) [34]. The accumulation of inflammatory mediators disrupts the coagulation–fibrinolysis balance, activates plasma coagulation cascades, impairs physiological anticoagulant function and leads to a prothrombotic state, ultimately causing thrombus formation. Hence, controlling inflammation through NF-κB is an essential strategy for mitigating cerebral ischemia. Our research findings indicate that pre-treatment with LYC exhibits good anti-inflammatory effects in laboratory studies (Figure 6). Whether pre-treatment with LYC can become a therapeutic strategy for IS requires further exploration.

## 5. Conclusions

In summary, our research indicates that LYC can prevent BV-2 cells from becoming polarized in response to LPS and from secreting inflammatory factors. Additionally, LYC can alleviate symptoms of cerebral ischemia in MCAO rats and reduce inflammation. We elucidate that LYC inhibits the activation of the NF-κB pathway, which is how it produces its anti-inflammatory effects. Therefore, LYC has potential in the pre-treatment of IS and awaits further clinical trials for validation.

## Figures and Tables

**Table 1:** List of primer sequences (Sangon Biotech, China).

Gene	Forward Primer	Reverse Primer
Mouse IL-1β	5′-GCAACTGTTCCTGAACTCA-3′	5′-CTCGGAGCCTGTAGTGCAG-3′
Mouse IL-6	5′-GAAAAGAGTTGTGCAATGGC-3′	5′-GTACTCCAGAAGACCAGAGGA-3′
Mouse TNF-α	5′-GCCACCACGCTCTTCTGTCTAC-3′	5′-GACGGCAGAGAGGAGGTTGACT-3′
Rat IL-1β	5′-GCACAGTTCCCCAACTGGTA-3′	5′-AAGACACGGGTTCCATGGTG-3′
Rat IL-6	5′-AGCGATGATGCACTGTCAGAA-3′	5′-GCATTGGAAGTTGGGGTAGGA-3′
Rat TNF-α	5′-GAGCACGGAAAGCATGATCC-3′	5′-TTTGGGAACTTCTCCTCCTTGT-3′
Mouse/Rat β-actin	5′-AGCCATGTACGTAGCCATCC-3′	5′-CTCTCAGCTGTGGTGGTGAA-3′

**Table 2:** Survival rates of rats following MCAO.

	Sham	MCAO	MCAO + LYC-1	MCAO + LYC-5
Total	6	9	9	9
Survival	6	6	6	6
Survival rate (%)	100%	66.7%	66.7%	66.7%

## Data Availability

Dataset available on request from the authors. The raw data supporting the conclusions of this ar-ticle will be made available by the authors on request.

## Supplementary Materials

The following supporting information can be downloaded at: https://www.mdpi.com/article/10.3390/brainsci15030290/s1. File S1: Immunofluorescence staining results of BV-2 cell survival.
