# Supplementary material for: Lycorine Pre-Treatment Alleviates Microglia Inflammation After Cerebral Ischemia by Inhibiting NF-κB Phosphorylation
Source: Brain Sci. 2025 Mar 9;15(3):290. doi: 10.3390/brainsci15030290 (PMC11939849; doi:10.3390/brainsci15030290)
Supplement: Supplementary file 1 [file brainsci-15-00290-s001.zip › brainsci-3474402-supplementary.pptx]

## Slide 1
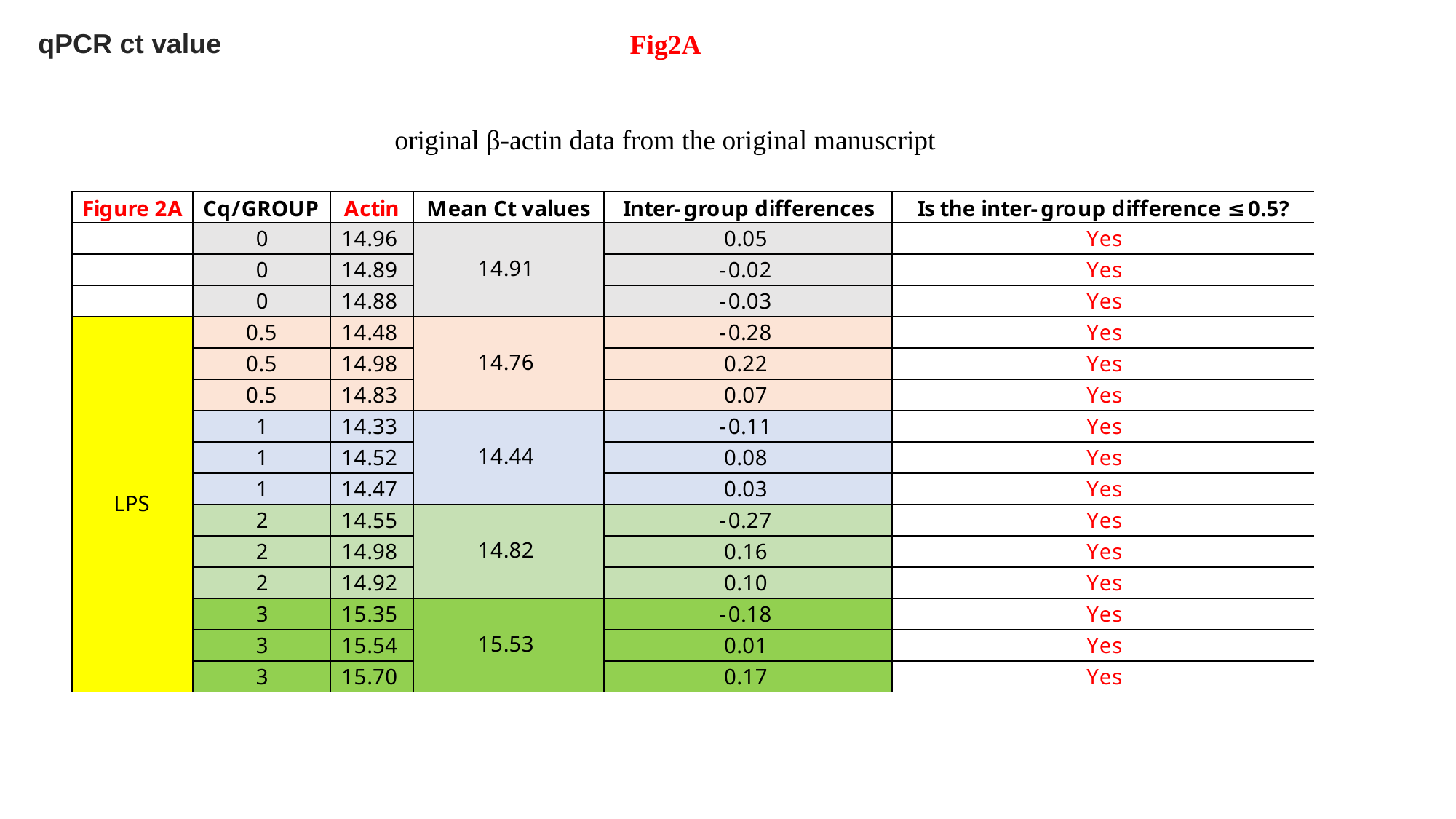

qPCR ct value
Fig2A
original β-actin data from the original manuscript

## Slide 2
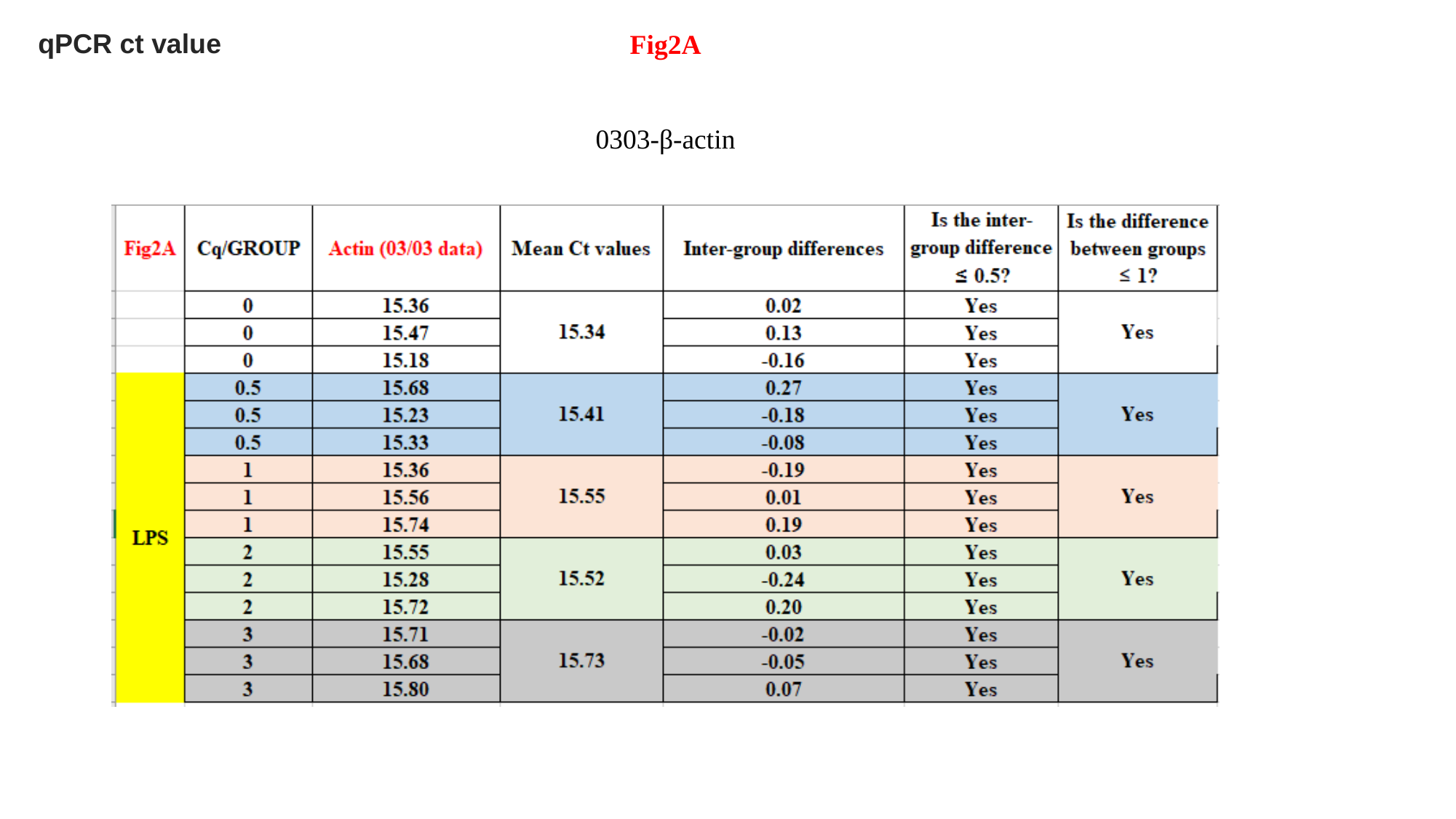

qPCR ct value
Fig2A
0303-β-actin

## Slide 3
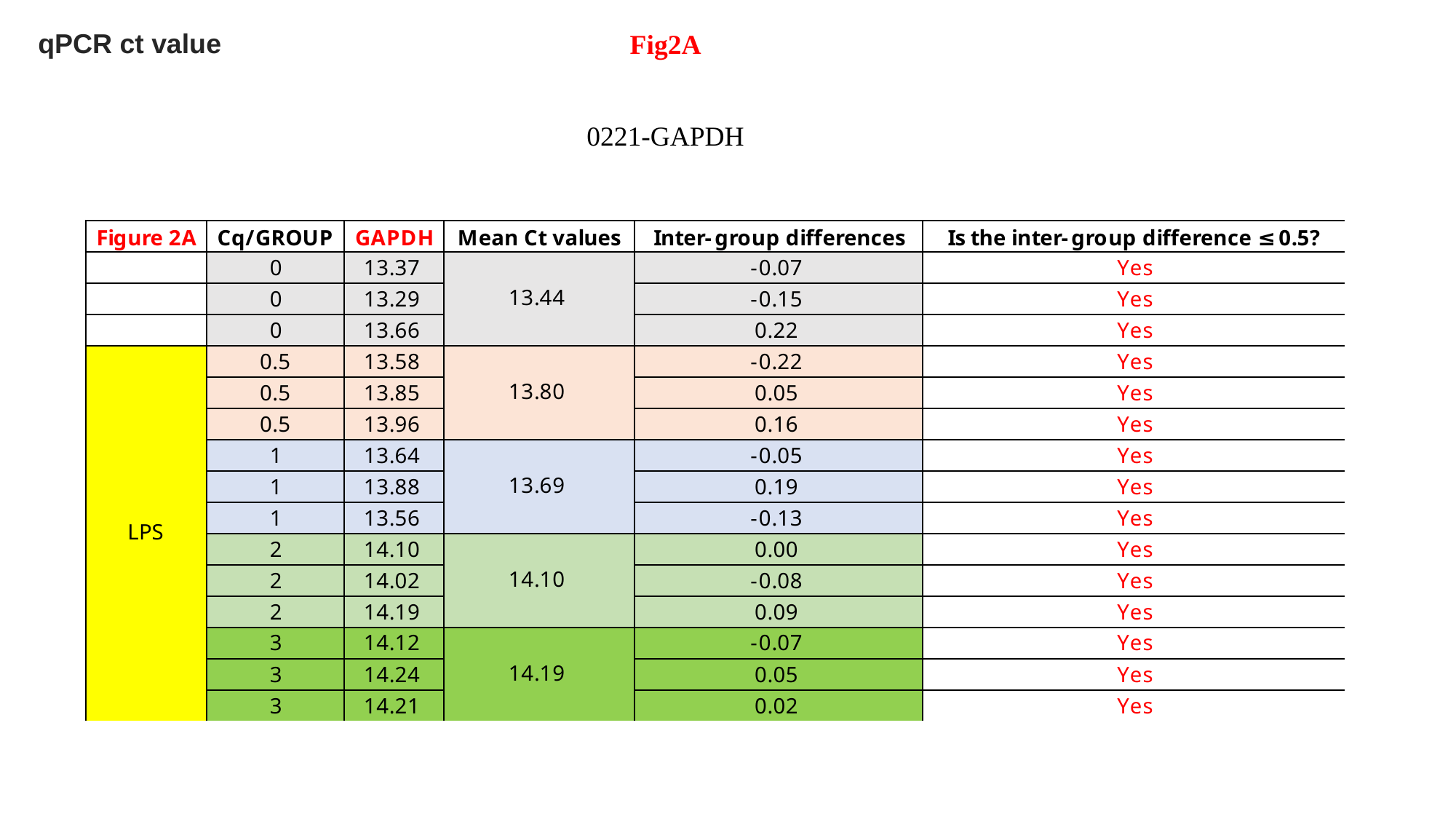

qPCR ct value
Fig2A
0221-GAPDH

## Slide 4
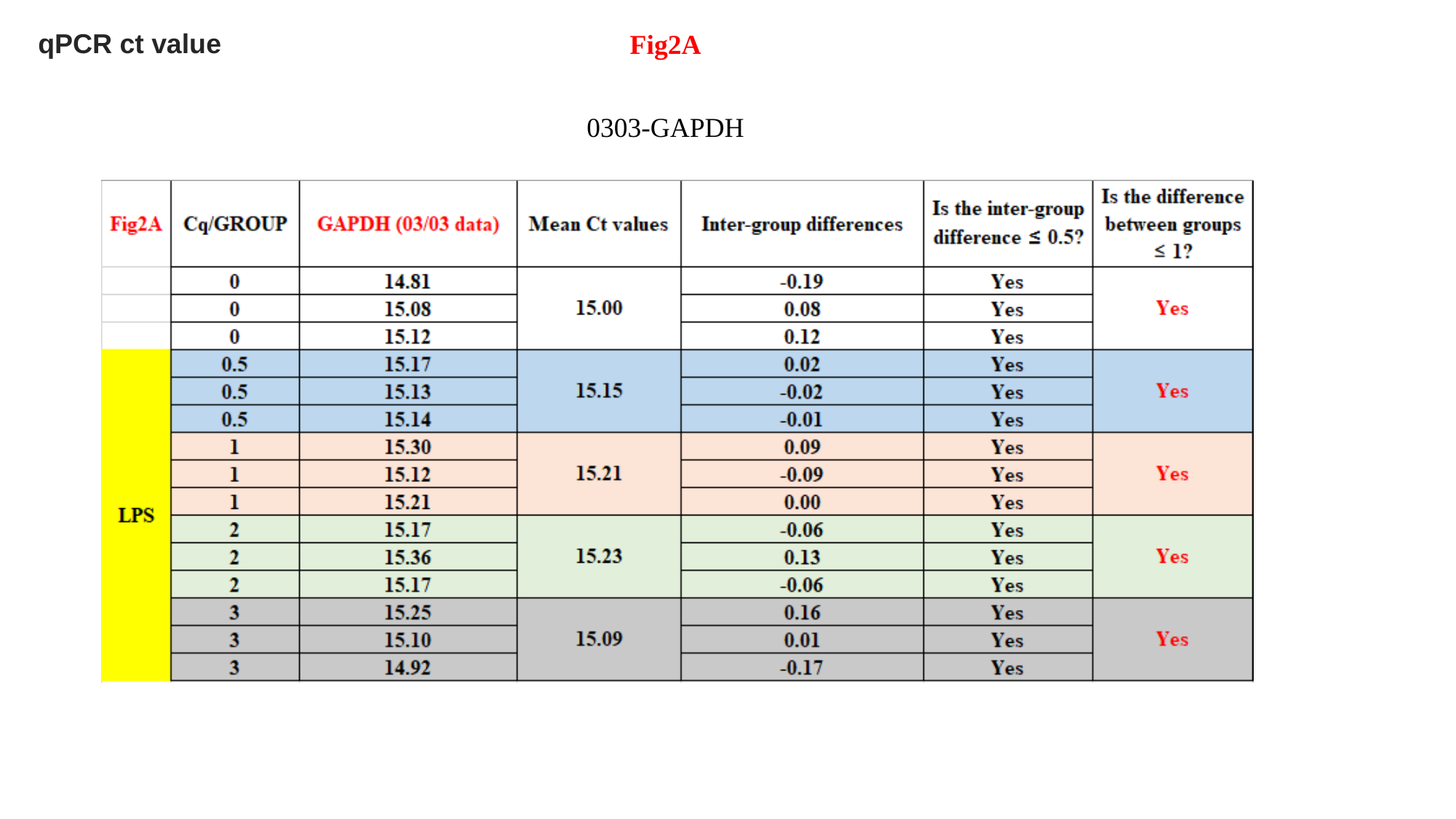

qPCR ct value
Fig2A
0303-GAPDH

## Slide 5
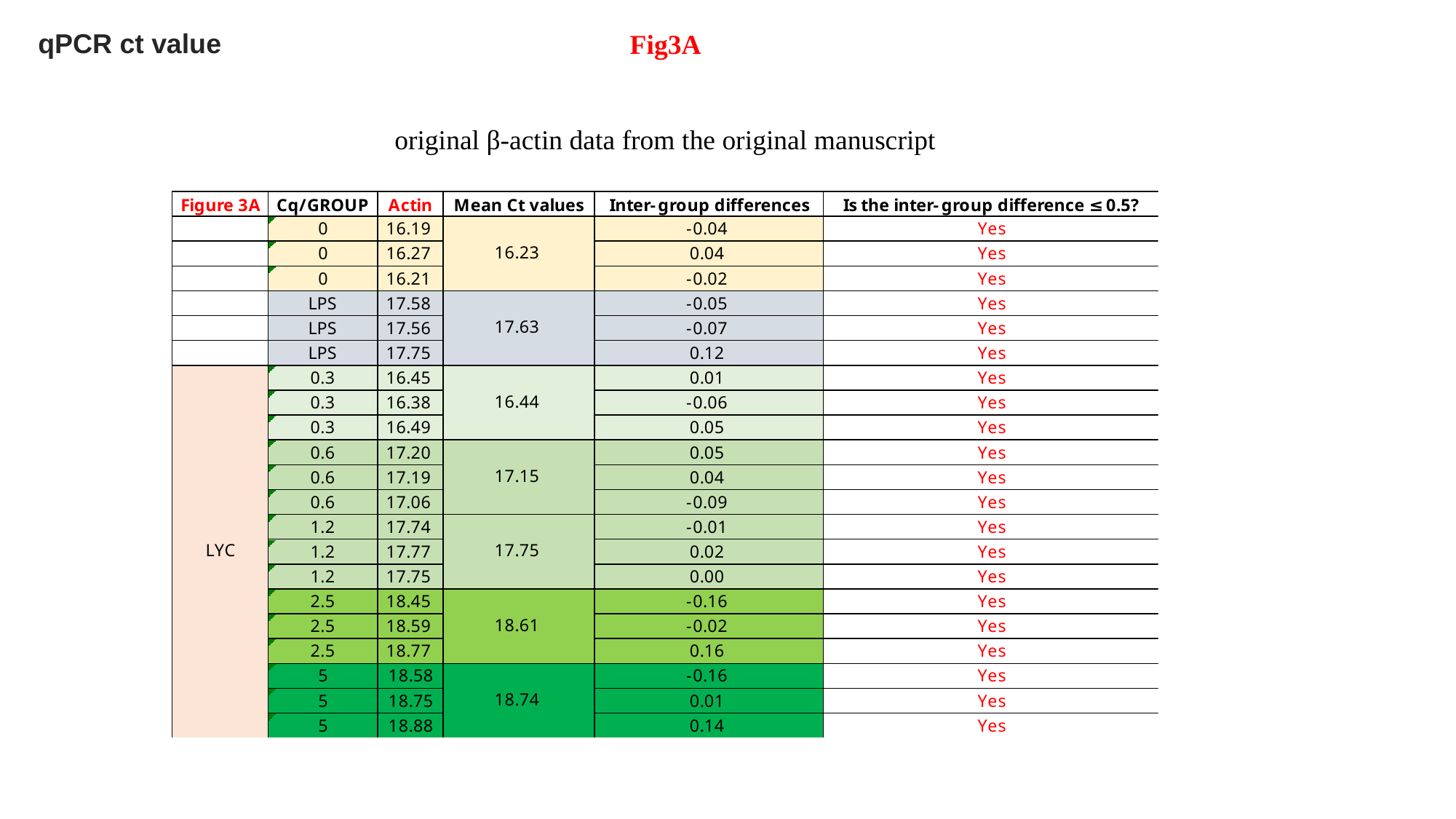

qPCR ct value
Fig3A
original β-actin data from the original manuscript

## Slide 6
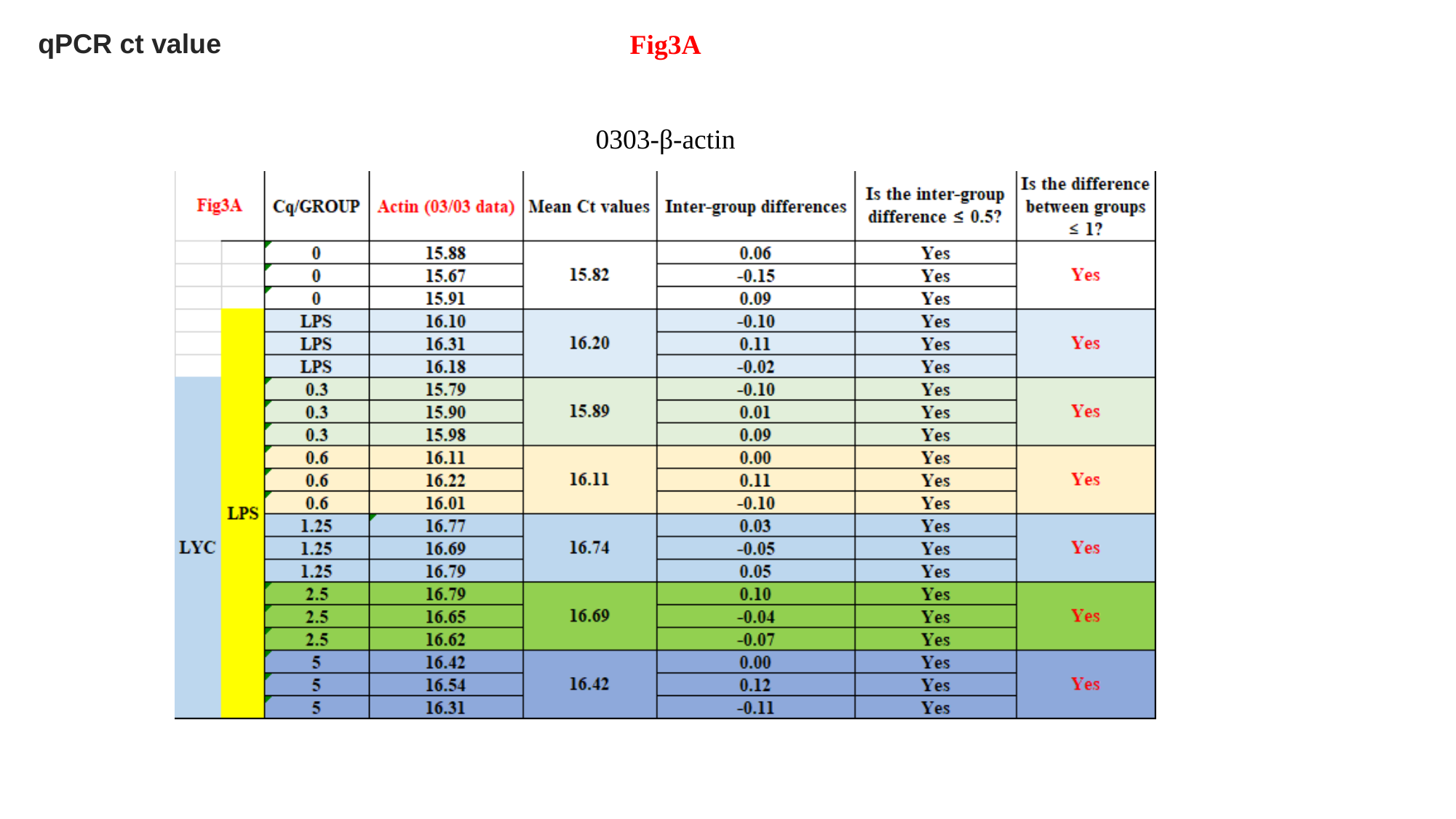

qPCR ct value
Fig3A
0303-β-actin

## Slide 7
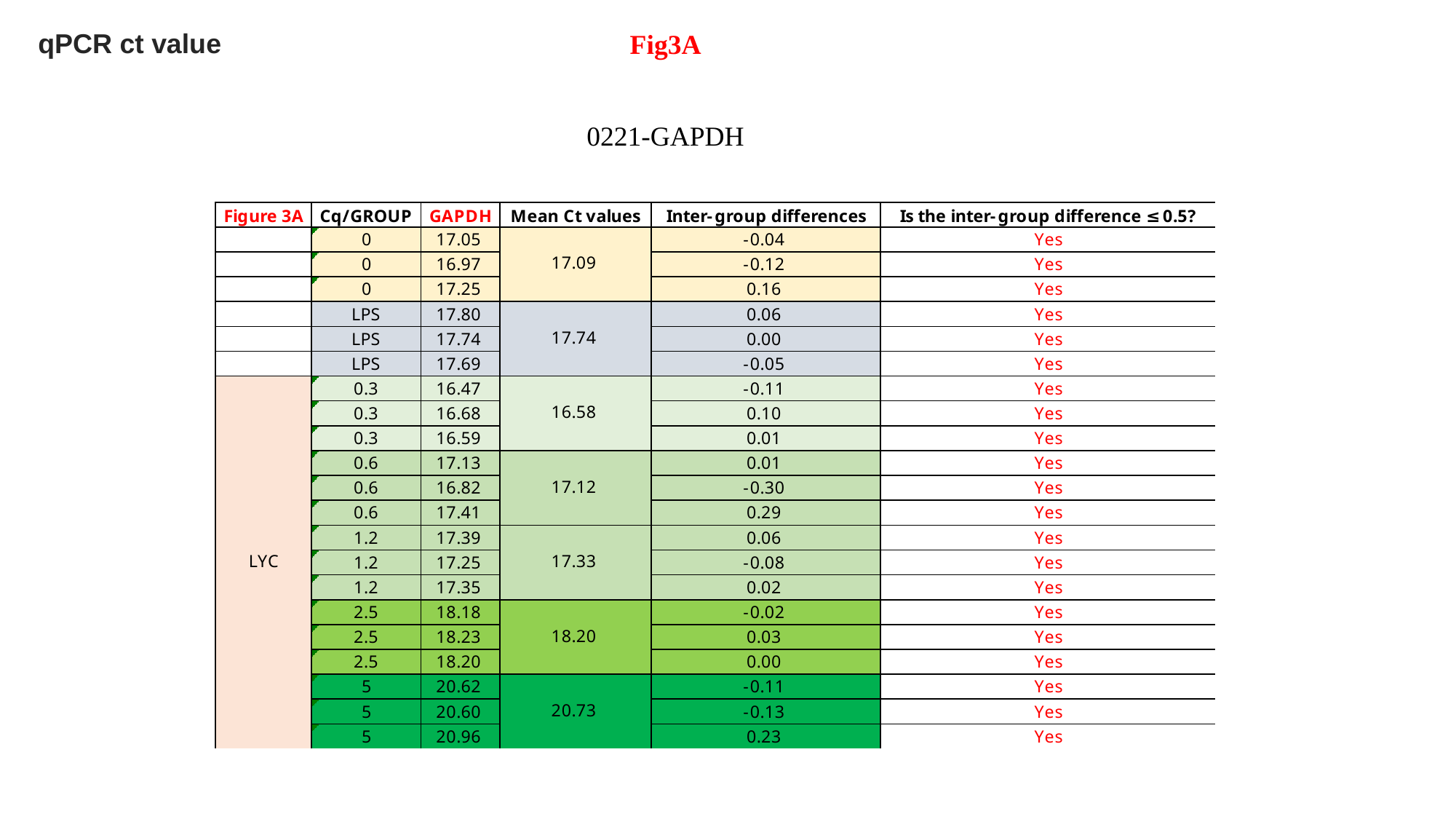

qPCR ct value
Fig3A
0221-GAPDH

## Slide 8
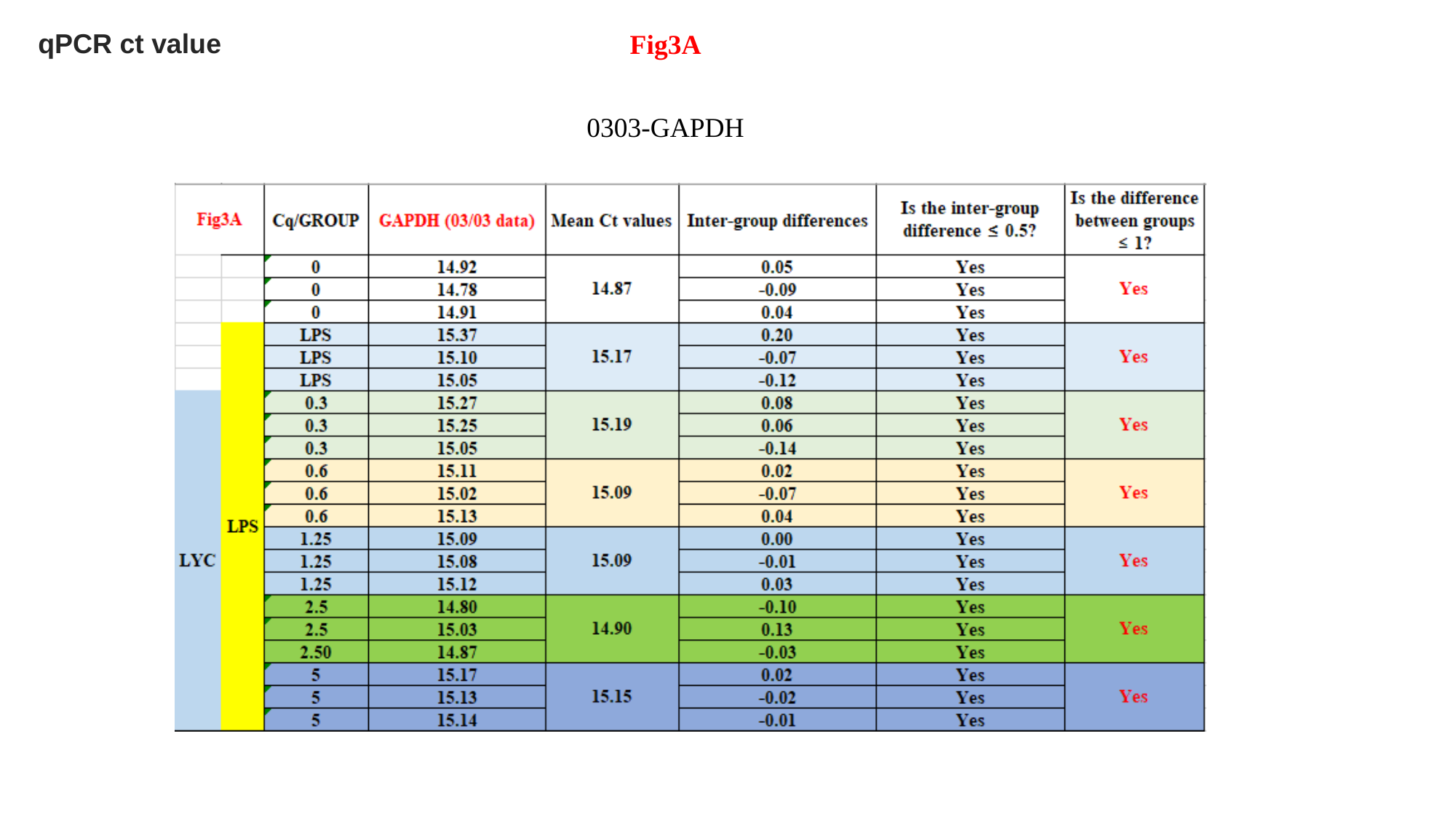

qPCR ct value
Fig3A
0303-GAPDH

## Slide 9
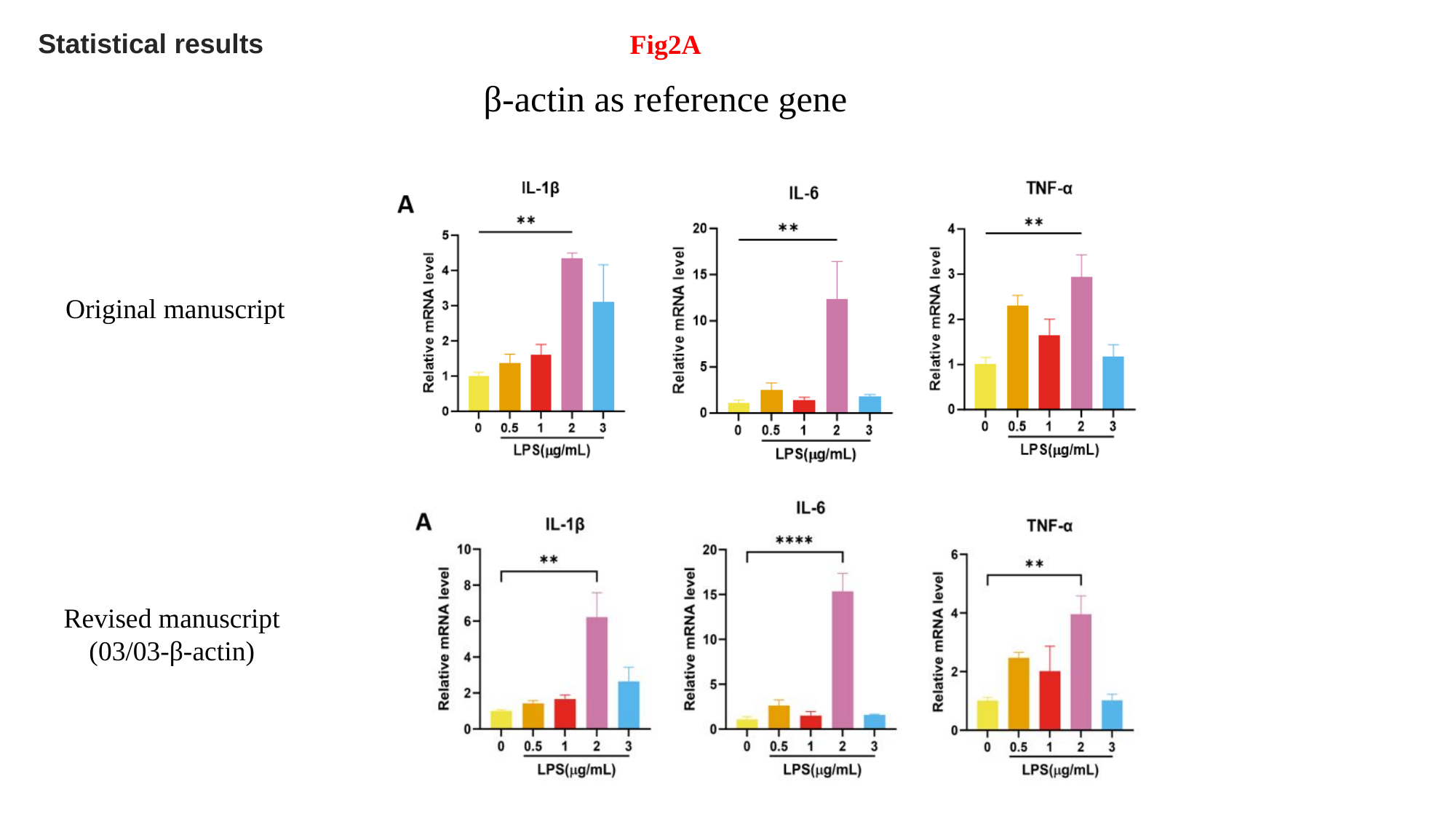

Statistical results
Fig2A
β-actin as reference gene
 Original manuscript
Revised manuscript
(03/03-β-actin)

## Slide 10
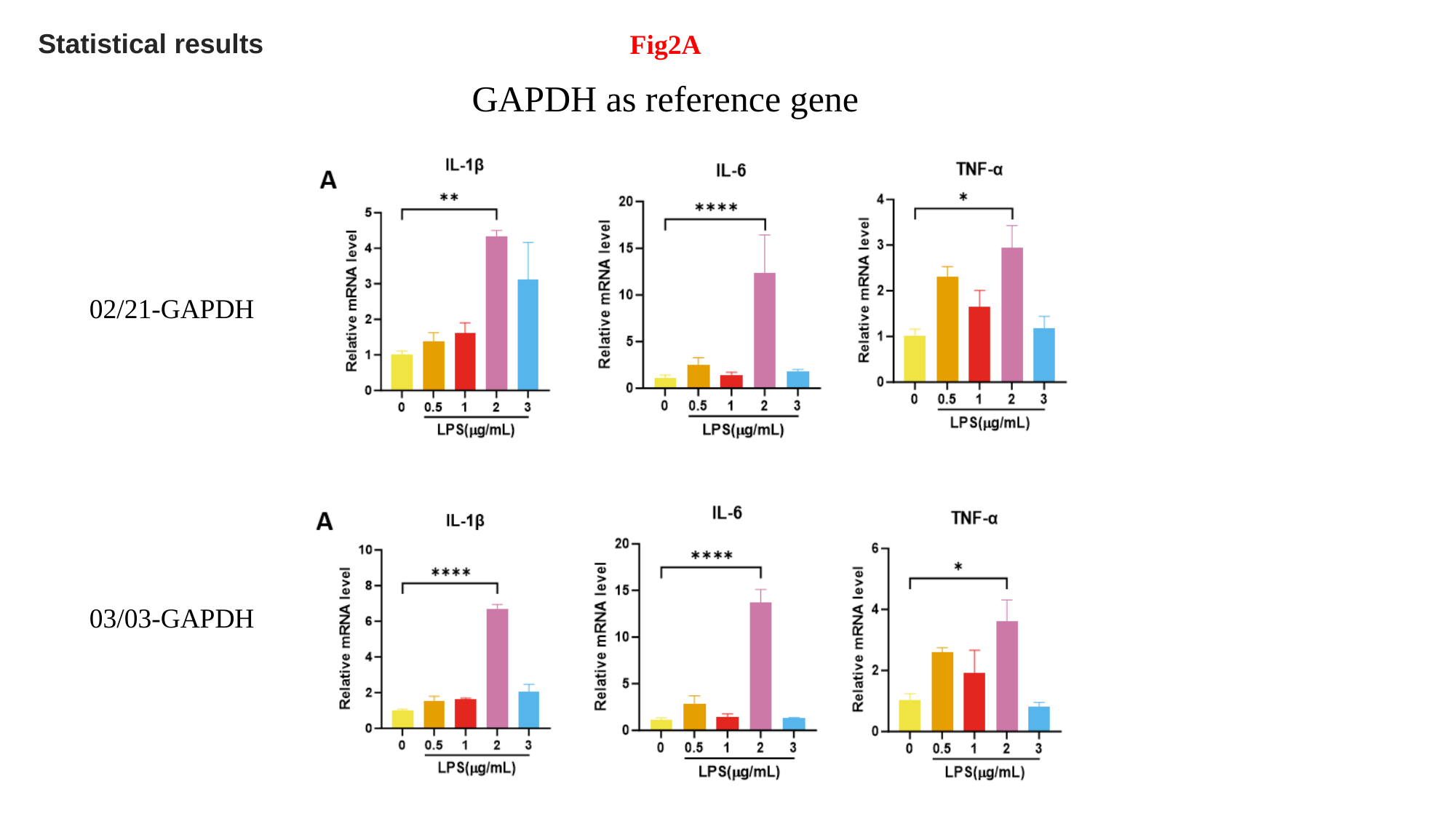

Statistical results
Fig2A
GAPDH as reference gene
02/21-GAPDH
03/03-GAPDH

## Slide 11
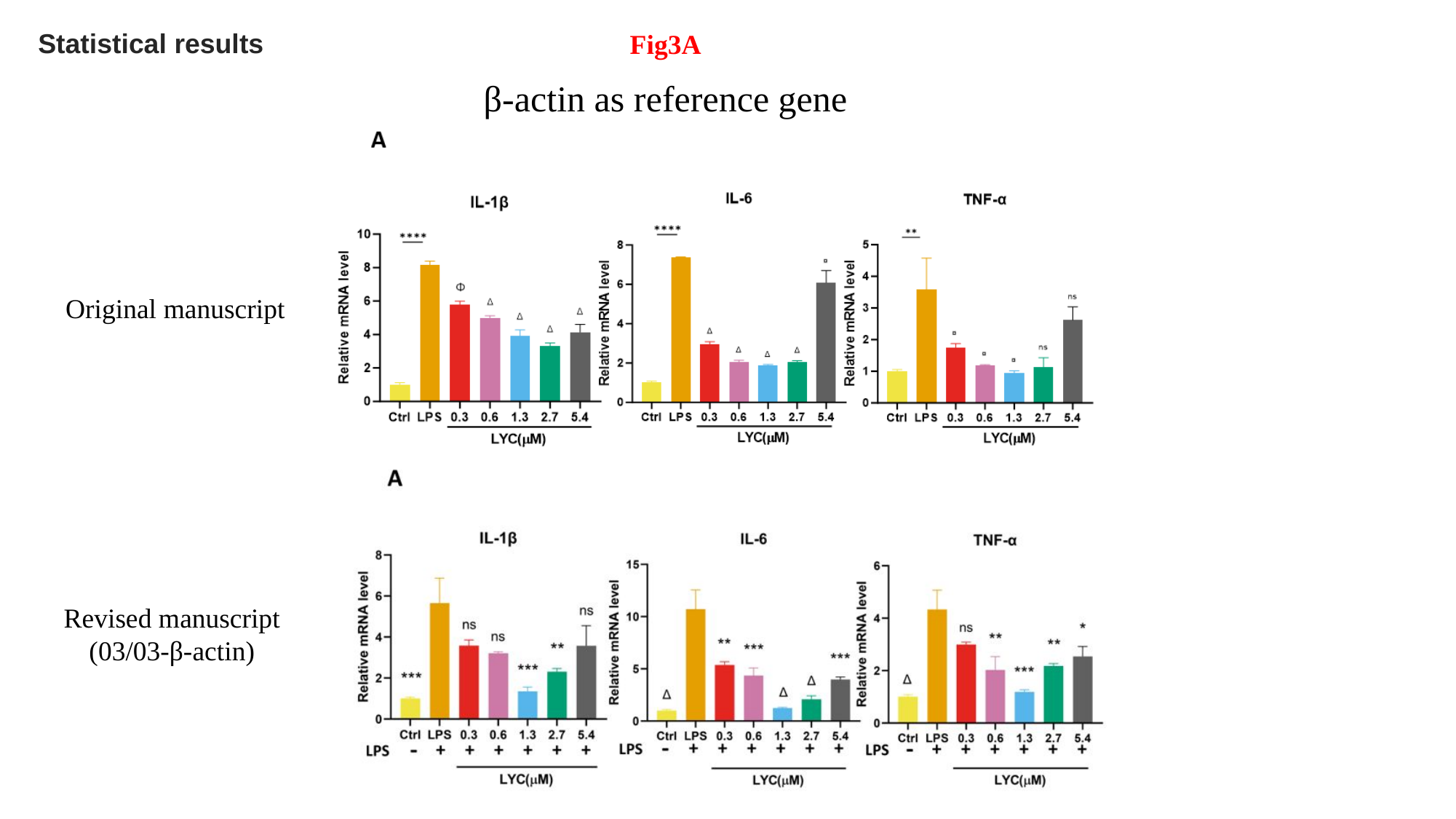

Statistical results
Fig3A
β-actin as reference gene
 Original manuscript
Revised manuscript
(03/03-β-actin)

## Slide 12
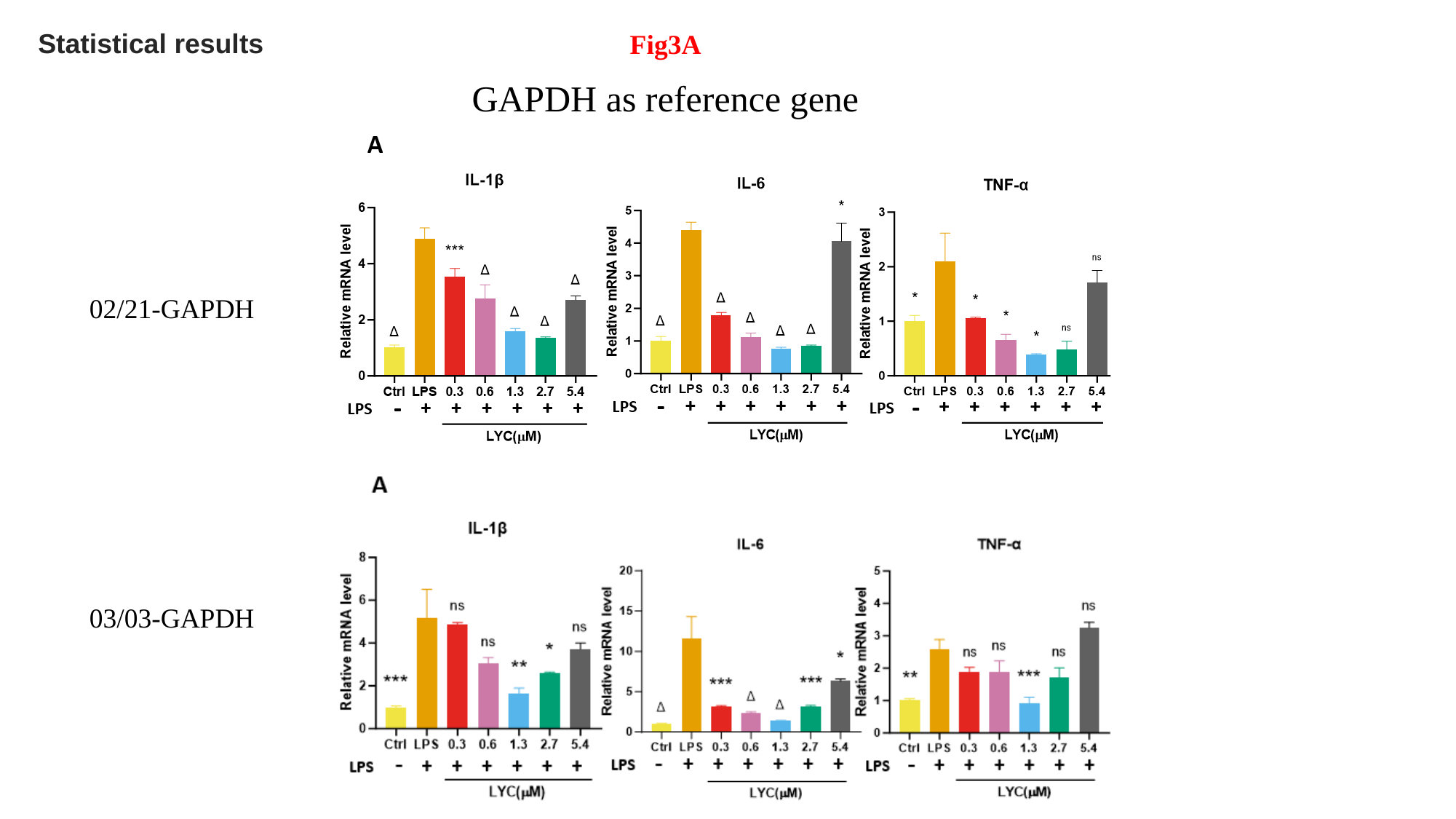

Statistical results
Fig3A
GAPDH as reference gene
02/21-GAPDH
03/03-GAPDH
